# DNA Methylation in the CYP3A Distal Regulatory Region (DRR) Is Associated with the Expression of CYP3A5 and CYP3A7 in Human Liver Samples

**DOI:** 10.3390/molecules29225407

**Published:** 2024-11-16

**Authors:** Joseph M. Collins, Danxin Wang

**Affiliations:** Department of Pharmacotherapy and Translational Research, College of Pharmacy, Center for Pharmacogenomics and Precision Medicine, University of Florida, Gainesville, FL 32610, USA; jcoll86@cop.ufl.edu

**Keywords:** cytochrome P450s, CYP3A4, CYP3A5, CYP3A7, DNA methylation, gene expression, distal enhancer

## Abstract

CYP3As are important drug-metabolizing enzymes in the liver. The causes for large inter-person variability in CYP3A expression/activity remain poorly understood. DNA methylation broadly regulates gene expression and the developmental transition from fetal CYP3A7 to adult CYP3A4, and CpG methylation upstream of the CYP3A4 promoter is associated with its expression. However, because non-promoter CYP3A regulatory regions remain largely uncharacterized, how DNA methylation influences CYP3A expression has yet to be fully explored. We recently identified a distal regulatory region (DRR) that controls the expression of CYP3A4, CYP3A5, and CYP3A7. Here, we investigated the relationship between CYP3A expression and the methylation status of 16 CpG sites within the DRR in 70 liver samples. We found significant associations between DRR methylation and the expression of CYP3A5 and CYP3A7 but not CYP3A4, indicating differential CYP3A regulation by the DRR. Also, we observed a dynamic reduction in DRR DNA methylation during the differentiation of induced pluripotent stem cells to hepatocytes, which correlated with increased CYP3A expression. We then evaluated the relative contribution of genetic variants, TFs, and DRR DNA methylation on CYP3A expression in liver samples. Our results reinforce the DRR as a CYP3A regulator and suggest that DNA methylation may impact CYP3A-mediated drug metabolism.

## 1. Introduction

The cytochrome P450s (CYPs) metabolize endogenous substrates and xenobiotics, including steroid hormones, carcinogens, toxins, and medications. Members of the CYP3A subfamily are highly abundant and crucial enzymes for the metabolism of xenobiotics, influencing nearly 50% of clinically used medications [1]. Four functional CYP3A genes, CYP3A4, CYP3A5, CYP3A7, and CYP3A43, are located in a gene cluster on chromosome 7q22.1. CYP3A4 is the most abundant CYP3A enzyme in adult livers, while the expression of CYP3A5 is distributed across several tissues and varies greatly due to a frequent genetic variant, CYP3A5*3, that is aberrantly spliced and a significant reduction in functional CYP3A5 [2]. CYP3A7 is primarily expressed during fetal development, and its expression is much lower in the adult liver. The final member, CYP3A43, is the least studied, as it shows low expression in the liver and extra-hepatic tissues, like the testes and prostate.

Large inter-person variability exists in CYP3A expression and activity, greatly affecting drug exposure and treatment outcomes [1]. Most *cis*-acting genetic polymorphisms for the CYP3A genes are regulatory variants that alter CYP3A expression, as coding variants are rare in these genes [3], except for CYP3A5*7, which is a frameshift mutation [4]. Variants instead typically alter expression by affecting splicing, such as the CYP3A5 genetic variants CYP3A5*3 and *6 [2] and CYP3A4*22 [5,6]. CYP3A7*1C contains a sequence that is identical to a CYP3A4 promoter fragment between −291 and −232 bp (relative to the ATG start site) and is associated with increased CYP3A7 expression [7]. However, the currently known CYP3A variants cannot fully explain the variation in CYP3A expression, which is heritable and not only driven by environmental interactions [8].

The CYP3As are regulated by numerous transcription factors (TFs), including liver-enriched TFs and nuclear hormone receptors, for example, Pregnane X Receptor (PXR/NR1I2), Constitutive Androstane Receptor (CAR/NR1I3), Hepatocyte Nuclear Factor 4 Alpha (HNF4A), Estrogen Receptor alpha (ESR1), etc. [9]. Notably, unliganded ESR1 is a master regulator for the expression of all CYP3As and other CYPs in the liver [10]. Combined, the expression of ESR1 and other TFs can explain a significant portion of the variability in CYP3A expression in the liver, specifically CYP3A4 (57%), CYP3A5 (34%), and CYP3A7 (14%) [9].

In addition to cis-acting genetic variants and TFs, epigenetic factors such as miRNAs [11,12], non-coding RNAs [13], histone modifications [14], and DNA methylation [15] also control CYP3A expression. DNA methylation and the histone code extensively crosstalk, establishing chromatin domains that control gene expression and genome integrity [16]. As such, DNA methylation is commonly profiled, as it can reveal regulatory dynamics and is easier to evaluate than the histone modifications. For example, the DNA methylation status of several CpG sites in the proximal promoters of CYP3A4 and CYP3A7 differ between prenatal and postnatal livers and correlate with changes in the developmental expression of these two enzymes [17]. Also, in adult liver samples, the methylation status of several CpG sites upstream of the CYP3A4 promoter is associated with its expression [15]. These studies highlight how DNA methylation at singular CpG sites instead of CpG islands near CYP3A promoters can regulate their expression.

While CYP3A7 is considered a fetal enzyme, its expression can also be observed in adult liver samples [18]. In contrast to CYP3A4 and CYP3A5, the variability in CYP3A7 is only moderately attributable to TF expression [9], indicating that other factors, like DNA methylation, may be controlling CYP3A7 expression in adults. In addition to promoters, the DNA methylation status of enhancers also modulates gene expression [19] and is known to control developmental timing [20]. Several proximal and distal CYP3A4 enhancers have been identified [21,22,23,24,25], and DNA methylation at these sites does not associate with CYP3A4 expression [15], but whether DNA methylation contributes to the expression of the other CYP3As has not been studied.

We recently identified a distal regulatory region (DRR) located downstream of CYP3A4 (~90 kb) and CYP3A7 (~40 kb) and upstream of CYP3A5 (~13 kb) [25,26]. CRISPR-mediated deletion of the DRR in Huh7 cells showed that it acts as a shared enhancer for CYP3A4, CYP3A5, and CYP3A7 but not CYP3A43. An investigation into the DRR revealed several potential CpG sites for DNA methylation; however, the influence of these sites on regulatory activity is unknown. Here, we tested the relationship between the methylation status of the DRR and the expression of the CYP3A genes.

## 2. Results

### 2.1. Methylation of the CpG Sites Within the DRR

**Figure 1:**
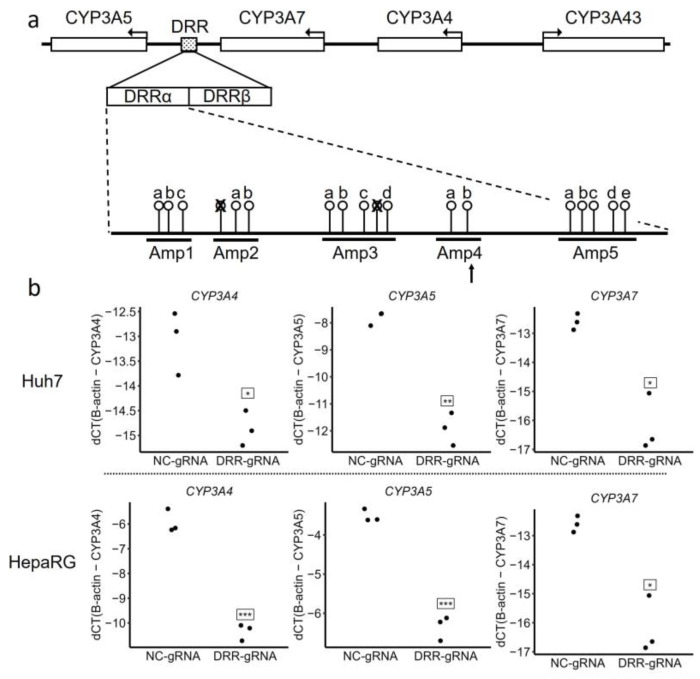
The DRR regulates CYP3A expression and contains several CpG sites. (**a**) The diagram shows the location of the DRR and the CpG sites within the 5′ end of the DRR. The PCR amplicons measured after bisulfite treatment are indicated. Two sites within Amp 2 and Amp 3 were excluded from the analysis. The arrow indicates that the CRISPRi gRNA target is located in Amp 4, specifically at chr7:99693627-99693646 (GRCh38.hg38). The letters above Amp1-Amp5 indicate CpG sites within each amplicon. (**b**) The effects of CRISPRi-mediated inhibition of the DRR on CYP3A expression. Plots show the dCT value for each tested gene (*n* = 3). NC-gRNA, non-target control gRNA; DRR-gRNA, gRNA targeting the DRR. Student’s *t*-test results: * *p* < 0.05, ** *p* < 0.01, *** *p* < 0.001.

To validate the role of the distal enhancer DRR in regulating the CYP3As, we used CRISPR interference (CRISPRi) to target and inhibit its activity [27]. Chromatin features indicative of enhancers (e.g., H3K4me1) suggest that the DRR is roughly 4 kb in length and can be divided into two subregions, DRRα and DRRβ (Figure 1a) [26]. gRNA was designed to target the region between DRRα and DRRβ to inhibit the activity of both subregions. Consistent with our previous results, DRR inhibition reduced the expression of CYP3A4, CYP3A5, and CYP3A7 in both Huh7 and HepaRG cells (Figure 1b), further illustrating that the DRR is a shared distal enhancer for the three CYP3A genes. The expression of CYP3A43 in Huh7 and HepaRG cells was too low to detect consistently, so it was not evaluated here.

Due to its potential to regulate three CYP3A genes, we investigated whether any CpG sites within the DRR could be methylated and alter its function. Only a few CpG sites exist in its ~2.5 kb 3′ portion, while 18 CpG sites were found within its ~1.5 kb 5′ segment (chr7:99,692,433–99,694,127 GRCh38/hg38) (Figure 1a). To test for potential methylation of these sites, we bisulfite-treated liver sample DNA and amplified the 5′ DRR region using five pairs of overlapping primers (Supplementary Table S1). Bisulfite treatment converts unmethylated cytosines (Cs) to thymines (Ts) but does not affect methylated Cs; thus, the ratio of C/T at each CpG site (measured with SNaPshot assays [28]) reflects the ratio of methylated/unmethylated Cs at a given CpG site. SNaPshot primers could not be designed for two sites in amplicons 2 and 3 due to their proximity to neighboring sites and were thus excluded (Figure 1a). We first screened ten samples to explore the methylation status of the remaining 16 CpG sites. Two sites within amplicon 4 were completely unmethylated in all ten samples. The other 14 had variable DNA methylation levels within the samples (Supplementary Figure S1) and also differed between samples, with interquartile ranges (IQRs) from 0.58 to 13.64 (Supplementary Table S2).

**Figure 2:**
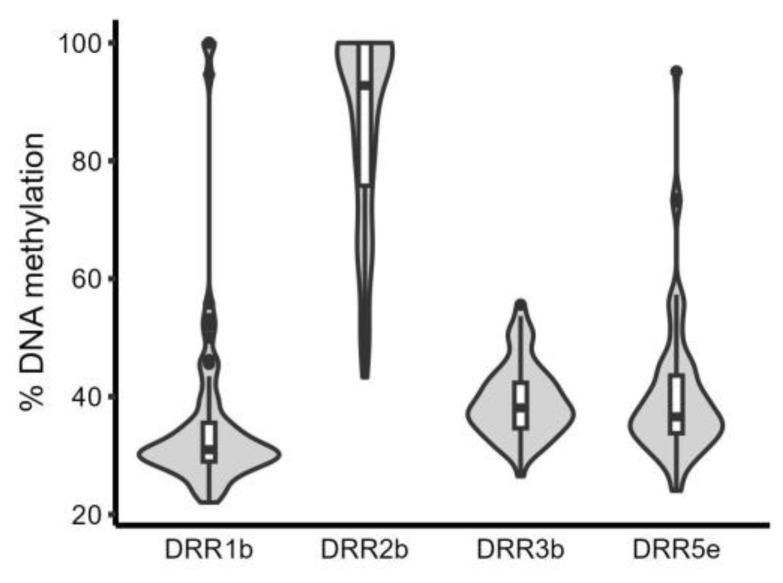
The cytosine methylation levels of sites 1b, 2b, 3b, and 5e in the 70 liver samples. Violin plots show the distribution of the percentage DNA methylation for each CpG site, with internal boxplots showing median and IQR and whiskers showing 1.5*IQR. The dots indicate outliers.

The methylation levels at some sites were highly correlated within samples. For example, the three sites within amplicon 1 (1a, 1b, and 1c) and one of the CpGs in amplicon 2 (2a) showed a strong correlation, as did the five sites within amplicon 5 (Supplementary Table S3). In contrast, the 2b CpG site was either not correlated or anti-correlated with the other CpG sites and also had the highest average methylation level (56%) and greatest IQR between individuals. Based on the exploratory results, we selected 1b, 2b, 3b, and 5e for further analysis in an additional 60 liver samples. They were chosen because 1b captured the trends of 1a,1b,1c, and 2a; 2b was variably methylated and not correlated with the other CpGs; 3b had the largest inter-person IQR of the CpGs in amplicon 3; and 5e captured the trends of the CpGs in amplicon 5, while also having the highest IQR. Table 1 shows the pairwise Pearson correlation coefficient of the methylation levels at these four sites in all 70 liver samples, which range from weak to moderately correlated (Table 1), in agreement with the exploratory results. The average DNA methylation levels were similar in the whole cohort compared to the smaller subset; however, site 2b showed higher average methylation when more samples were tested (92% vs. 56%; Figure 2). There were no associations between the DNA methylation level of the four CpGs and age, race, or sex, except 5e, which was associated with age and had a 1% reduction in methylation per year (*p* = 0.011).

### 2.2. Associations Between the DNA Methylation of the DRR and the Expression of the CYP3As

We used the Pearson correlation to test the correlation between DNA methylation at the specific CpG sites and expression of the CYP3As. DNA methylation at CpG site 1b was negatively correlated with expression levels of CYP3A4 and CYP3A43, while DNA methylation at site 2b is positively correlated with the expression of CYP3A5 (*p* < 0.05) (Table 2). Since TFs and genetic variants also regulate the expression of the CYP3As, we used stepwise multiple linear regression to evaluate the contribution of these factors to the expression of the CYP3As. The candidate predictors included were CYP3A4*22 (rs35599367), CYP3A5*3 (rs776746), CYP3A7*1C (rs11568824), seven TFs (ESR1, NR1I3, NR1I2, FOXA2, RXRA, HNF4A, ARNT, and AHR) known to affect CYP3A expression [9], and the DNA methylation level at the four CpG sites (Table 3). For CYP3A4, TFs were the strongest predictors, together explaining ~70% of its variability between samples, and CYP3A4*22 explained an additional ~2%, thereby together explaining over 72% of the variability in CYP3A4 expression, consistent with our previous report [9]. After adjusting for the TFs and CYP3A4*22, DNA methylation at site 1b was no longer associated with CYP3A4 expression (Table 3). For CYP3A5, TFs and CYP3A5*3 were the main predictors, explaining 34% and 30% variability in CYP3A5 expression, respectively. After adjusting for TFs and CYP3A5*3, increased DNA methylation at site 1b remained significantly associated with increased expression of CYP3A5 and could explain an additional 2% of the overall variability in CYP3A5 expression. Two TFs (RXRA and ESR1) and CYP3A7*1C were the main predictors for CYP3A7 expression, explaining 18.8% and 22.6% of the variability, respectively. DNA methylation was associated with decreased (site 3b) or increased (site 5e) expression of CYP3A7, which together explained an additional 4% of the variability in CYP3A7. Like CYP3A4, the expression of CYP3A43 was strongly associated with the expression levels of TFs. There was no significant association between DRR DNA methylation and the expression of CYP3A43, consistent with our previous results showing no DRR-mediated regulation of CYP3A43 [26].

### 2.3. Inter-Person Variability in the Composition of CYP3A Pools

**Figure 3:**
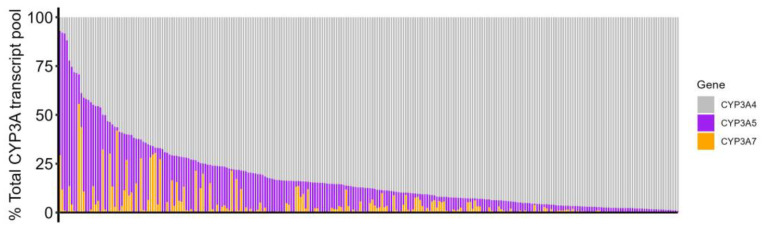
The composition of the CYP3A pools in 246 liver samples. CYP3A4, CYP3A5, and CYP3A7 are each shown as their percentage of the total CYP3A pool. Each column shows a different sample.

We then evaluated the relative expression of CYP3A4, CYP3A5, and CYP3A7 in 246 liver samples and found large inter-person variability in the composition of each sample’s CYP3A pool (Figure 3). On average, the relative amount of each CYP3A family member in the total CYP3A pool agreed with previously published reports, with CYP3A4 having the highest expression (82% of the total CYP3A content), followed by CYP3A5 (14%) and CYP3A7 (4%), consistent with the protein expression of the CYP3As in the liver [29]. However, several samples (19/260 or 7.3%) had low expression of CYP3A4 (contributing to <50% of the CYP3A pool), and so CYP3A5 and CYP3A7 became the primary CYP3A transcript content in these samples. At the extreme, CYP3A5 represented 91% of the total CYP3A content in one sample, and CYP3A7 reached 56% in another (Supplementary Table S4a). Similar results were observed in the subset of 70 samples with DNA methylation data (Supplementary Figure S2 and Table S4b). Using these data, we evaluated which factors (TFs, variants, and DRR DNA methylation) were associated with the relative expression of each CYP3A in the CYP3A pools. The results were consistent with our analysis of each CYP3A expression in Table 3, except that the percentage of CYP3A4 was also affected by the CYP3A5*3 genotype (Table 4). This result makes sense, as individuals with CYP3A5*3 had 8-fold-less CYP3A5 overall [9], thereby increasing the relative amount of CYP3A4 in the CYP3A pool. DNA methylation status was also associated with the percentage of CYP3A5 (site 1b) and CYP3A7 (site 5e) in the CYP3A pool.

### 2.4. Changes in the DRR DNA Methylation Status During iPSC-to-Hepatocyte Differentiation

**Figure 4:**
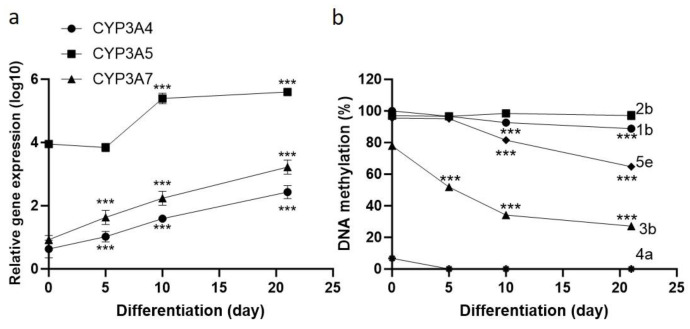
CYP3A expression and CpG methylation changes in the DRR during iPSC-to-hepatocyte differentiation. iPSCs were subject to hepatocyte differentiation, and cells were harvested at day 0 (iPSC), day 5 (definitive endoderm), day 10 (progenitor cells), and day 21 (hepatocyte-like cells) for measuring (**a**) CYP3A gene expression and (**b**) DNA methylation of sites 1b, 2b, 3b, 4a, and 5e. *** Compared to the iPSC stage (day 0), ANOVA with Dunnett’s post-test, *** *p* < 0.001.

We used iPSC-to-hepatocyte differentiation as a cellular model to test whether DNA methylation changed at the DRR during the differentiation process and whether it correlated with CYP3A expression. Cells were harvested at multiple time points to capture the differentiation process: day 0 (iPSC), day 5 (definitive endoderm stage), day 10 (progenitor cells), and day 21 (hepatocyte-like cells, HLC). The expressions of albumin and HNF4A, two top HLC markers [30], were undetectable in the iPSCs and became detectable on day 21 (Ct values of 26 and 24 for HNF4A and albumin, respectively), indicating the successful differentiation of iPSCs to HLCs. Consistently, the expression levels of CYP3A4 and CYP3A7 (also HLC markers [30]) were nearly undetectable in the iPSC stage and steadily increased throughout differentiation, with their highest expression occurring at the HLC stage (Figure 4a and Supplementary Table S5). Conversely, CYP3A5 was expressed at the iPSC stage (~8000–10000-fold higher than either CYP3A4 or CYP3A7), and its expression did not increase until the progenitor cell stage, where it also increased like CYP3A4 and CYP3A7 and reached levels comparable to liver samples at the HLC stage (Supplementary Table S5). All of the tested DRR CpG sites were highly methylated (80–100%) in the iPSCs (Figure 4b), except for site 4a, which was unmethylated, as observed in the liver samples. Site 2b remained completely methylated throughout the differentiation process, and site 1b showed small (~10%) but significant reductions in DNA methylation levels at days 10 and 21. In contrast, the methylation levels at sites 3b and 5e drastically reduced over the differentiation process (Figure 4b), coinciding with increased expression of the CYP3As.

## 3. Discussion

In this study, we measured the methylation status of 14 CpG sites within the DRR enhancer that regulates the expression of CYP3A4, CYP3A5, and CYP3A7. We then tested whether the CpG methylation of four representative CpG sites was associated with the expression of these three CYP3As. Our results showed significant associations between the expression levels of CYP3A5 and CYP3A7 and the methylation status of several CpG sites within the DRR after adjusting for the genetic variants and the expression of several TFs. To our knowledge, this is the first study to test the association between DNA methylation and the expression CYP3A5 and CYP3A7 while also evaluating the relative contributions of genetic variants, TFs, and DNA methylation on the expression and composition of CYP3A pools in adult human liver samples.

The CYP3As metabolize numerous endogenous and exogenous compounds and are regulated by numerous TFs (see the review [31]). Several constitutive or inducible distal enhancers have been reported for CYP3A4 [21,22,23,24]. The methylation status of the CpG sites in these regions (8–10 kb upstream of the CYP3A4 promoter) is not associated with CYP3A4 expression after adjusting for TFs [15]. Likewise, our results showed no association between the expression of CYP3A4 and CpG methylation in the DRR enhancer (Table 3), which regulates constitutive CYP3A4 expression [26]. Only the two CpG sites located ~1400 bp upstream of the CYP3A4 promoter are known to be associated with CYP3A4 expression to date [15]. Our previous work shows that the CYP3As are regulated by multiple enhancers [25], and we believe that there remain more CYP3A enhancer regions regulating their expression (unpublished), each with potential CpG methylation that contributes to their function.

The DRR contains more CpG sites (15 per 1 kb vs. <10 per 1 kb) than the previously identified CYP3A4 enhancers [15], and the CpG density is higher in the DRR compared to the genome-wide average (3 per 544 bp) [32]. Excluding site 2b, the methylation levels were low for all sites tested (average <50%) (Table S2), and two sites (4a and 4b) were unmethylated in all samples. Interestingly, sites 4a and 4b overlap with an enhancer RNA (eRNA) listed in the eRNAbase (Sample-01-011810198) [33], which is expressed in the liver and is predicted to target CYP3A4, CYP3A5, and CYP3A7. Consistently, CpG sites surrounding the eRNA also had low methylation levels (~20%), with small inter-person variability (IQR < 5). Enhancers that transcribe eRNAs have lower DNA methylation [34], and eRNAs play critical roles in directing enhancer activity to their target genes [35]. Therefore, reduced methylation across this region of the DRR may be necessary for its regulatory function.

CRISPRi targeting of the DRR decreased the expression of three CYP3As (Figure 1a), similar to previous results using CRISPR-mediated deletion of the DRRβ [26]. The CRISPRi gRNA used here targeted a region between the DRRα and DRRβ, likely inhibiting both subregions due to CRISPRi’s ability to inhibit regions up to 1kb [36]. However, CpG methylation of the DRR appears to affect each CYP3A uniquely. While the expression of CYP3A4 was not associated with any differences in the methylation status of the DRR, CYP3A5 and CYP3A7 were, and both had different CpG sites associated with their expression (Table 3). These results are consistent with our previous report showing that two SNPs within the DRR regulate either CYP3A4 or CYP3A5, but not both, indicating differential regulation of the CYP3As by the DRR. Notably, CRISPR-mediated deletion of the DRRα did not affect CYP3A5 expression [26], while our results showed that the CpG site 1b, located within the DRRα, was associated with increased CYP3A5 expression. Although the underlying mechanism is not clear, it seems likely that differential CpG methylation alters the binding of TFs or other chromatin remodelers at the DRR, not only in the proximity of the CpG sites but also in the flanking regions [37,38], thereby influencing its capacity to regulate the CYP3As. In liver samples and HepG2 cells, ENCODE data reveal that the entire DRR is a TF-binding hotspot [26], including for known liver TFs (e.g., HNF41A, HNF4A, RXRA, FOXA1, and FOXA2), all of which show strong preferences for unmethylated binding sites in HepG2, based on methmotif.org [39]. How DNA methylation of the DRR specifically alters its function is an intriguing future direction, as it will likely give broader insight into CYP3A regulation.

The timing of CYP3A expression throughout cellular differentiation agrees with the final tissue distribution of each CYP3A. The expression of CYP3A4 and CYP3A7 was nearly undetectable in the iPSCs and did not increase until differentiation into definitive endoderm. The definitive endoderm gives rise to liver and intestinal cells, both of which are the primary tissues where these two genes are expressed, suggesting that their expression fate may be established early in the differentiation process. In contrast, the expression of CYP3A5 in the iPSCs was relatively high, which agrees with its wider tissue distribution in hepatic and extra-hepatic tissues, based on GTEx [40]. Therefore, despite these three genes having similar roles in the cell and being located within a gene cluster, their expression is uniquely controlled. The three CpG sites (1b, 3b, and 5e) that showed significant associations with the expression of CYP3A7 or CYP3A5 were highly methylated in the iPSCs, the levels of which drastically reduced as hepatic differentiation proceeded (Figure 4b). At these sites, methylation was inversely correlated with the expression of the CYP3As, with the DNA methylation steadily decreasing as the cells differentiated and CYP3A expression increased (Figure 4 and Supplementary Table S5). These results suggest that the reduction in methylation across the DRR may coincide with hepatic differentiation, establishing a regulatory landscape that promotes the expression of the CYP3As. In contrast, DNA methylation at the 1b and 5e sites was positively associated with the expression of CYP3A5 and CYP3A7, respectively. As development and differentiation are known to be controlled by the interplay between enhancers and TFs [41], and since DNA methylation alters TF binding affinities [38,42], methylation at these two sites may alter TF binding, resulting in the expression of specific CYP3A isoforms. Therefore, although a regulatory region may influence the expression of several genes, it seems likely that variation within a regulatory region may not affect all target genes equally.

Our results showed significant differences in the composition of CYP3A transcript pools between liver samples. In general, CYP3A4 constituted the majority of the CYP3A pool in most samples, although the combined proportion of CYP3A5 and CYP3A7 exceeded 50% in 19 samples (7% of the cohort) (Figure 3). Fourteen of these nineteen samples were from African American donors who carried CYP3A5*1, indicating that CYP3A5*1 carriers can produce CYP3A5 at levels similar to CYP3A4, in agreement with previous studies [2,4]. Overall, CYP3A7 minimally contributed to the total CYP3A pool (on average, 4%), but 14 samples (5.3%) had CYP3A7 content ranging from 20 to 55%, and therefore CYP3A7 may significantly impact overall CYP3A metabolism in these individuals. CYP3A7*1C was the main factor associated with high CYP3A7 expression, and the 14 CYP3A7*1C samples in our cohort typically had higher-than-average CYP3A7 expression. Still, there were many non-CYP3A7*1C samples with relatively high CYP3A7 expression (Supplementary Figure S3), and 27 that had more than 2-fold-higher CYP3A7 expression than the average, suggesting that there are additional factors contributing to the persistence of the fetal CYP3A7 in adults. Our results support DNA methylation at the DRR as one such factor, suggesting that variation in epigenetic features at the DRR may alter the expression of the CYP3As throughout development. Owing to the high homology between CYP3A4, CYP3A5, and CYP3A7, they share many common substrates, although with different affinities [43], and thus, the potential for the drug metabolism of CYP3A substrates by CYP3A5 and CYP3A7 should not be ignored, particularly in those individuals who have lower CYP3A4 expression. This is especially true for medications favored by either CYP3A5 or CYP3A7. For example, tacrolimus is primarily metabolized by CYP3A5, and CYP3A5 genotyping is recommended for its dosing [44]; and a recent screen identified 23 CYP3A7-specific substrates, including the antihypertensive medication fenoldopam [43], raising the possibility that variable CYP3A7 expression could affect fenoldopam exposure. Here, we identified DRR CpG site methylation as an additional factor influencing the expression of CYP3A5 and CYP3A7, with each site explaining ~2% total variability, similar to the amount of CYP3A4 variability explained by the genetic variant CYP3A4*22 (Table 3). Therefore, factors that modify the dynamic expression of these three CYP3A genes (e.g., DRR methylation levels) could affect the CYP3A-related metabolism of relevant medications, particularly in individuals with low expression of CYP3A4.

A limitation of this study is that it focused on a singular CYP3A enhancer and may not have captured effects occurring in other regulatory regions. However, except for the DRR, no other enhancers have been identified for CYP3A5 and CYP3A7, and CYP3A4 is only significantly associated with methylation in one proximal enhancer [15]. To fully understand the regulatory landscape of the CYP3As, it will be important to identify all CYP3A regulatory regions and evaluate both genetic and epigenetic variations in their function.

In summary, we found significant associations between CpG methylation within the DRR and the expression of CYP3A5 and CYP3A7. Future studies that focus on a comprehensive evaluation of the impacts of genetic and epigenetic variation across the CYP3A regulatory landscape will help to better explain the observed variability in the expression of the CYP3As and elucidate the causes for the ‘missing heritability’ of CYP3A metabolism.

## 4. Materials and Methods

### 4.1. Liver Sample Preparation

A total of 246 liver samples were obtained from the Cooperative Human Tissue Network (CHTN). The demographic information of the liver donors was previously reported [9]. A subset of the samples with available DNA (*n* = 70) was used to measure DNA methylation. For ease, the demographic information of this subset is reported in Supplementary Table S6. The University of Florida Institutional Review Board approved the study.

### 4.2. CRISPRi and Cell Culture

The lentiviral-based vector (pLV hU6-sgRNA hUbC-dCas9-KRAB-T2a-Puro) was a gift from Charles Gersbach (Addgene plasmid # 71236; http://n2t.net/addgene:71236, accessed on 01/October/2024; RRID:Addgene 71236). Guide RNAs (gRNAs) were designed using the CRISPick online tool [45,46], and sequences are in Supplementary Table S1. The gRNAs were cloned into the CRISPRi vector using Golden Gate Assembly Kit (New England Biolabs, Ipswich, MA, USA) according to the manufacturer’s protocol and a published protocol [47] with some modifications. All constructs were sequenced for fidelity. Lentiviral particles were produced via co-transfection of 2.3 µg CRISPR construct, 1.1 µg pvSV-G, and 1.7 µg psPAX2 into 90% confluent HEK-293T cells in 6 cm dishes using Lipofectamine 3000 (Life Technologies, Carlsbad, CA, USA). HEK293T cells were grown in DMEM/F12 (Gibco, 11320-033, Waltham, MA, USA) supplemented with 10% fetal bovine serum (FBS) (Gibco, A5256701, Waltham, MA, USA) and penicillin and streptomycin (Gibco, 5140122, Waltham, MA, USA). All cell lines were grown in a humidified incubator at 37 °C with 5% CO_2_. After six hours, the medium was replaced with fresh medium containing 1% bovine serum albumin (BSA). Forty-eight hours later, the supernatant was harvested and concentrated 30-fold with Lenti-X Concentrator (Takara Bio, San Jose, CA, USA). The pvSV-G was a gift from Akitsu Hotta (Addgene plasmid # 138479; http://n2t.net/addgene:138479, accessed on 01/October/2024; RRID:Addgene_138479, and the psPAX2 was a gift from Didier Trono (Addgene plasmid # 12260; http://n2t.net/addgene:12260, accessed on 01/October/2024; RRID:Addgene_12260).

Huh7 cells were grown in DMEM (Corning, 10-615-CV, Corning, NY, USA) supplemented with 10% FBS (Gibco, A5256701, Waltham, MA, USA) and penicillin and streptomycin (Gibco, 5140122, Waltham, MA, USA). Huh7 cells were seeded into 24-well plates to achieve 90% confluence. The following day, cells were transduced using 25 µL of viral particles and TransDux MAX™ (System Biosciences, Palo Alto, CA, USA). Forty-eight hours later, cells were detached via 0.25% trypsin (Gibco, 25200056, Waltham, MA, USA), and each 24-well transduction was split into three wells of a 48-well plate in medium containing 1 µg/mL puromycin. Cells were then grown for another ten days in medium containing puromycin; untransduced cells did not survive puromycin selection beyond the first two days.

HepaRG^®^ cells were purchased from Biopredic International (Saint Grégoire, France) and were expanded and maintained according to the manufacturer’s protocols. For transduction, undifferentiated HepaRG cells were grown for one week after seeding from freeze stocks and then passed into 12-well plates at ~90% confluence. Cells were transduced with 50 µL viral particles the following day, using TransDux MAX™ (System Biosciences, Palo Alto, CA, USA). Forty-eight hours later, cells were detached via 0.25% trypsin (Gibco, 25200056, Waltham, MA, USA), and each 12-well transduction was split into three wells of a 24-well plate in medium containing 1 µg/mL puromycin. The cells were then maintained in HepaRG^®^ growth medium for two weeks, followed by two weeks in HepaRG^®^ differentiation medium, per the standard manufacturer’s protocols. Total RNA was prepared from Huh7 and HepaRG^®^ cells using RNA miniprep kits (Zymo Research, Irvine, CA, USA).

The qScript Ultra Flex kit (VWR International, Radnor, PA, USA) with a blend of oligo d(T) and random hexamers synthesized cDNA from total RNA. qPCR measurements were conducted on a Quantabio Q real-time PCR instrument (VWR International, Radnor, PA, USA). Gene expression was measured using PowerUp™ SYBR green (Applied Biosystems, Foster City, MA, USA). Relative expression was obtained through β-actin normalization. CRISPRi cell-line qPCR data analysis was conducted in R version 4.3.3 with tidyverse v1.3.2 [48] and ggpubr v0.5 [49]. Student’s *t*-test was performed in base R to compare dCT values.

### 4.3. Human iPSC-to-Hepatocyte Differentiation

The human iPSC cell line SCTi003 (isolated from a healthy donor) was purchased from StemCell (Seattle, WA, USA). iPSCs were cultured in Laminin-521-coated 6-well plates with mTeSR plus medium and were then transferred to 24-well plates for hepatocyte differentiation using the STEMdiff Hepatocyte kit according to the StemCell (Seattle, WA, USA) protocol. Cells were harvested on days 0, 5, 10, and 21 for RNA and DNA preparation.

For iPSC-derived cells, cDNA was synthesized with a combination of gene-specific primers and oligo d(T), and gene expression was measured using the SYBR green-based method (see Supplementary Table S1 for primers). The relative expression of each gene was calculated using the following formula: expression level of the tested gene = antilog2(mean cycle-threshold (Ct) value of β-actin—mean Ct value of tested gene) × 10^8^.

### 4.4. Measuring DNA Methylation

DNA methylation of the DRR was determined using the single-nucleotide extension (SnaPshot) assay after bisulfite treatment of DNA, as described previously [28]. Sodium bisulfite conversion of the DNA was achieved using the EZ DNA methylation kit (Zymo Research, Irvine, CA, USA). Five pairs of methylation-specific PCR primers (Supplementary Table S1) were designed using Bisulfite Primer Seeker provided by Zymo Research (https://www.zymoresearch.com/pages/tools, accessed on 01/October/2024). These primers were used to amplify the DRR region (chr7:99,692,433-99,694,127, GRCh38/hg38) after bisulfite treatment, and the PCR products were then subjected to SNaPshot assays to determine the ratio of C/T or G/A conversion, which represents the ratio of methylated/unmethylated cytosine at a given CpG site. As described below, the ratios were converted to % DNA methylation from standard curves for each CpG site.

The DRR (1694 bp) was PCR amplified from liver DNA (without bisulfite treatment). A portion of the PCR product was treated with CpG methyltransferase (M.SssI, New England Biolabs, Ipswich, MA, USA) to methylate all cytosine residues (C5) within the double-stranded dinucleotide recognition sequence (5′..CG..3′). The fully methylated DNA was mixed with unmethylated DNA (untreated PCR products) to produce samples representing DNA methylation levels of 0%, 25%, 40%, 75%, and 100%. These mixtures were then subjected to bisulfite treatment, PCR amplification, and SNaPshot assays (as described above) to determine the conversion ratios of C/T or G/A. Standard curves were then established by plotting the ratios against the percentage of methylation DNA in the mixture (Supplementary Figure S4). The percentage methylation of each tested sample was then extrapolated using the standard curve for each CpG site.

### 4.5. Liver Gene Expression, Genotyping, and Data Analysis

Liver gene-expression data were obtained from a previously published study [9]. β-Actin expression was used as an internal control. The relative expression of each gene was calculated using the following formula: the expression level of the tested gene = antilog2(mean Ct value of β-actin—mean Ct value of tested gene) × 10^6^. After the log10 transformation, the expression level of all genes followed a normal distribution.

CYP3A4*22 and CYP3A5*3 were genotyped using the OpenArray genotyping platform (QuanStudio 12 K Flex System, Life Technologies, Carlsbad, CA, USA) according to the manufacturer’s protocol. We used rs11568824 to represent CYP3A7*1C, which was genotyped using Snapshot Assays [28], with primers shown in Supplementary Table S1.

Statistical analysis was performed using Minitab 21 software or GraphPad Prism 10. Pairwise Pearson correlation was used to test the relationship between variables. A multiple linear regression model was used to test the association between DNA methylation and gene expression. We used forward, stepwise regression to select the best predictors in the multiple regression models with an α = 0.25 to enter.

## 5. Conclusions

We identified several CpGs in a distal regulatory region (DRR) and investigated the relationship between their DNA methylation status and the expression and composition of three CYP3As in a human liver cohort. Our results showed significant associations between the methylation status of several DRR CpG sites and the expression of CYP3A5 and CYP3A7 but not CYP3A4. Overall, our findings indicate that the DRR has unique regulatory control on each of the three CYP3As and suggest that factors affecting DNA methylation may also impact CYP3A metabolism.

## Figures and Tables

**Table 1:** Correlation between DNA methylation levels at CpG sites 1b, 2b, 3b, and 5e.

CpG Site	1b.	2b	3b
2b	r = 0.222, *p* = 0.064		
3b	r = 0.295, *p* = 0.013	r = 0.272, *p* = 0.022	
5e	r = 0.574, *p* = 2.04 × 10^−7^	r = 0.05, *p* = 0.681	r = 0.428, *p* = 2.16 × 10^−4^

**Table 2:** Correlation between expression of CYP3As and DNA methylation levels at CpG sites 1b, 2b, 3b, and 5e.

CYP3A Gene	CpG Site			
	1b	2b	3b	5e
*CYP3A4*	**−0.442**	0.036	−0.029	−0.156
*CYP3A5*	−0.109	**0.214**	−0.164	−0.201
*CYP3A7*	−0.156	0.137	−0.11	0.118
*CYP3A43*	**−0.34**	0.123	0.224	−0.09

The table shows the correlation coefficient, r, of each pair. Numbers in bold, *p* < 0.05.

**Table 3:** The association between the expression levels of the CYP3As and TFs, genotypes, and DNA methylation in human liver samples determined by multiple linear regression analysis.

Log(Gene)	Predictors	Estimate	*p*-Value	Variability Explained	Total R-sq
Log(CYP3A4)	ESR1	0.958	4.4 × 10^−14^	69.71% (All TFs)	72.27%
	ARNT	0.335	0.064		
	RXRA	−0.412	0.021		
	AHR	0.474	0.058		
	CYP3A4*22	−0.743	0.011	2.56%	
Log(CYP3A5)	ESR1	0.444	1.47 × 10^−5^	34.18% (All TFs)	67.56%
	NR1I2	0.423	0.011		
	HNF4A	−0.531	0.006		
	PPARA	0.437	0.004		
	Black Ancestry	0.315	0.011	1.01%	
	CYP3A5*3	−0.352	5.68 × 10^−6^	30.29%	
	1b DNA methylation	0.789	0.028	2.08%	
Log(CYP3A7)	RXRA	0.598	0.023	18.82% (All TFs)	45.60%
	ESR1	0.452	0.005		
	CYP3A7*1C	0.816	9.46 × 10^−5^	22.69%	
	3b DNA methylation	−3.021	0.026	4.09%	
	5e DNA methylation	1.863	0.031		
Log(CYP3A43)	ESR1	0.912	1.88 × 10^−10^	72.18% (All TFs)	72.18%
	AHR	1.249	9.38 × 10^−5^		
	ARNT	−0.635	0.006		
	RXRA	−0.664	0.006		
	HNF4A	0.58	0.037		

**Table 4:** The association between the composition of total CYP3A pools and TFs, genotype, and DNA methylation in human liver samples determined by multiple linear regression analysis.

Log(%Gene)	Predictors	Estimate	*p*-Value	Variability Explained	Total R-sq
Log(%CYP3A4)	ESR1	1.586	7.59 × 10^−9^	47.90%	52.27%
	RXRA	−0.109	0.03		
	CYP3A5*3	0.054	0.008	4.37%	
Log(%CYP3A5)	ESR1	−0.343	5.45 × 10^−4^	13.14%	58.42%
	FOXA2	0.436	0.013		
	CYP3A4*3	−0.361	1.10 × 10^−7^	42.78%	
	1b DNA methylation	0.852	0.033	2.50%	
Log(%CYP3A7)	RXRA	0.598	0.023	13.76%	39.49%
	ESR1	0.452	0.005		
	CYP3A7*1C	0.816	9.46 × 10^−5^	23.39%	
	5e DNA methylation	1.9	0.018	2.34%	

## Data Availability

The original contributions presented in the study are included in the article/Supplementary Materials; further inquiries can be directed to the corresponding author/s.

## Supplementary Materials

The following supporting information can be downloaded at https://www.mdpi.com/article/10.3390/molecules29225407/s1, Figure S1: Cytosine methylation of 16 CpG sites within the DRR in 10 representative samples; Figure S2: The composition of CYP3A pools in the 70 liver samples that also have DNA methylation data; Figure S3: Histogram showing the distribution of CYP3A7 expression in 246 liver samples; Figure S4: DNA methylation standard curves for the 14 CPG sites within the DRRα; Table S1: Sequence of primers used in this study; Table S2: Basic statistics of the DNA methylation levels of the 16 CpG sites in 10 samples; Table S3: Correlation between the DNA methylation levels of the 14 CpG sites in 10 samples; Table S4: The composition of the CYP3A pools in the 246 (a) or 70 (b) liver samples; Table S5: Changes in the expression levels of the CYP3As during iPSC to hepatocyte differentiation; Table S6: Demographics of liver donors measured for DNA methylation at the DRR.
