# Supplementary material for: DNA Methylation in the CYP3A Distal Regulatory Region (DRR) Is Associated with the Expression of CYP3A5 and CYP3A7 in Human Liver Samples
Source: Molecules. 2024 Nov 16;29(22):5407. doi: 10.3390/molecules29225407 (PMC11596782; doi:10.3390/molecules29225407)
Supplement: Supplementary file 1 [file molecules-29-05407-s001.zip › molecules-3267940-supplementary.pdf]

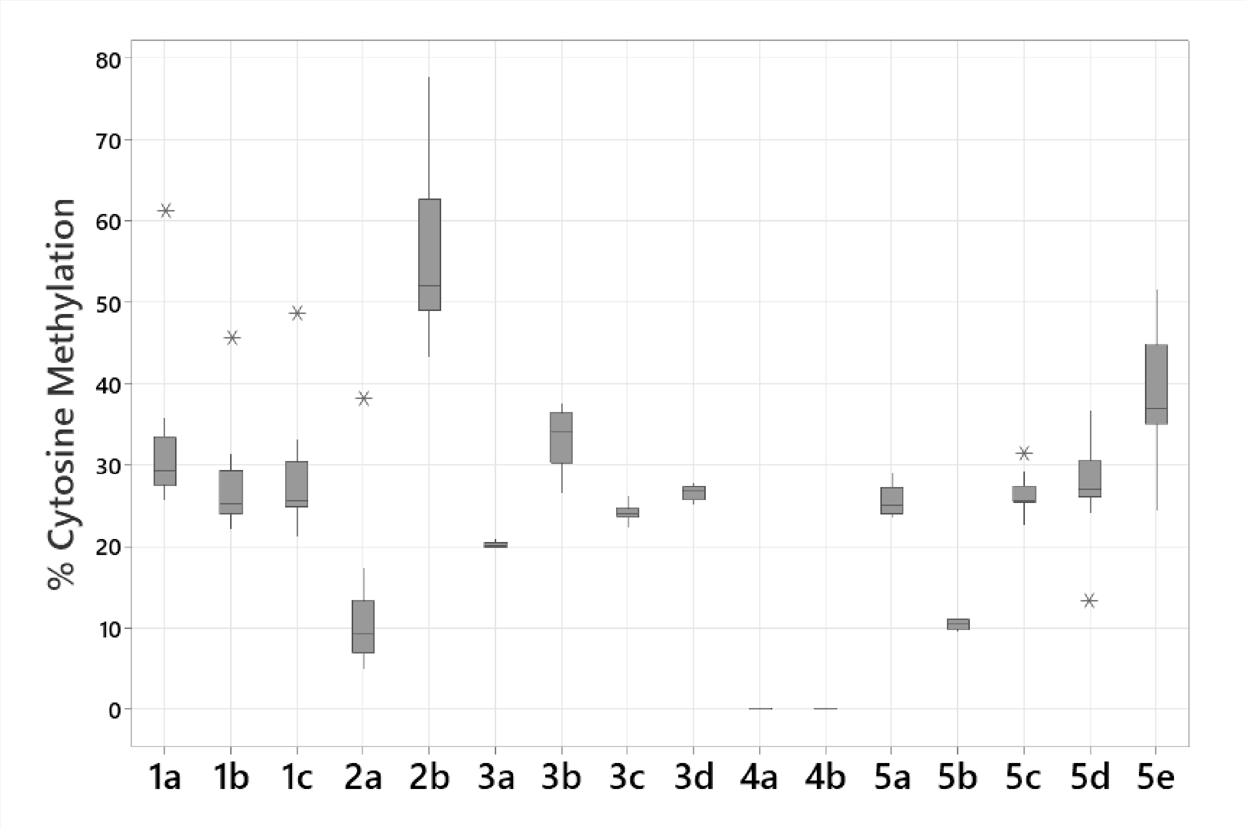

Figure S1. Cytosine methylation of the 16 CpG sites within the DRR in 10 representative samples. The lower and upper ends of the boxes show the 25th and 75th percentiles and horizontal lines show the mean percentage of cytosine methylation levels. The whiskers show the minimum and maximum values. Stars indicate the outliers.

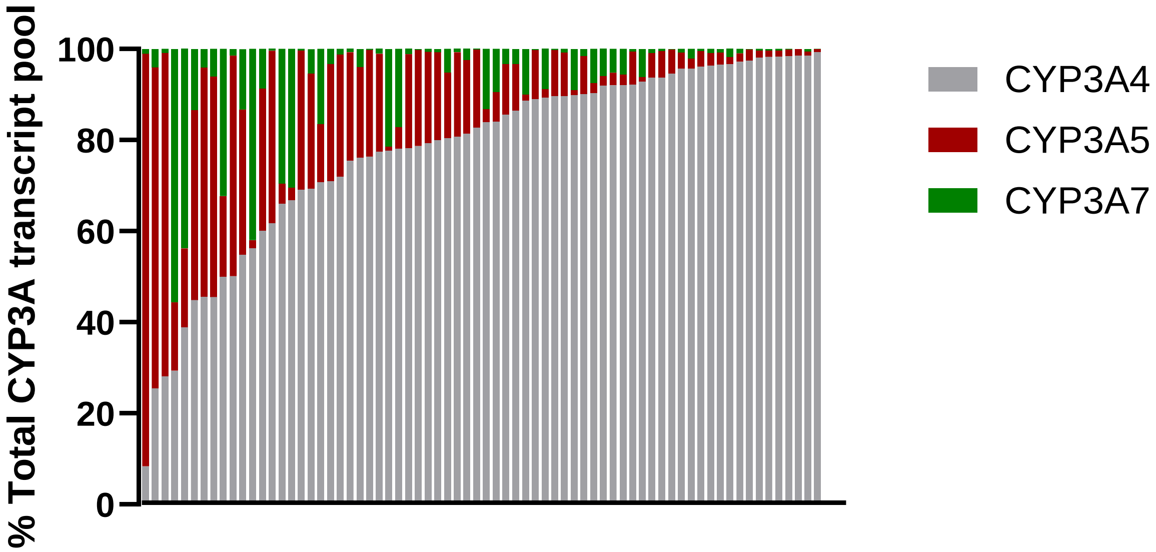

Figure S2. The composition of CYP3A pools in the 70 liver samples that also have DNA methylation data. The levels of CYP3A4, CYP3A5, and CYP3A7 are expressed as the percentage of the total CYP3A pools. Each vertical bar represents a different sample.

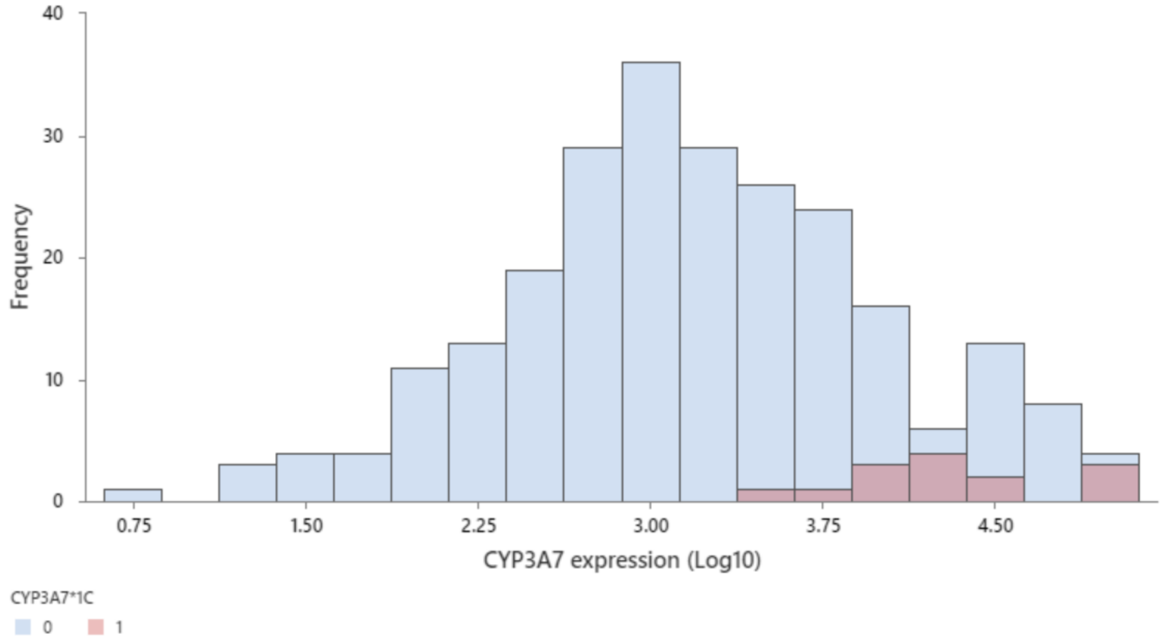

Figure S3. Histogram showing the distribution of CYP3A7 expression in 246 liver samples. The red color indicates CYP3A7*1C carriers.

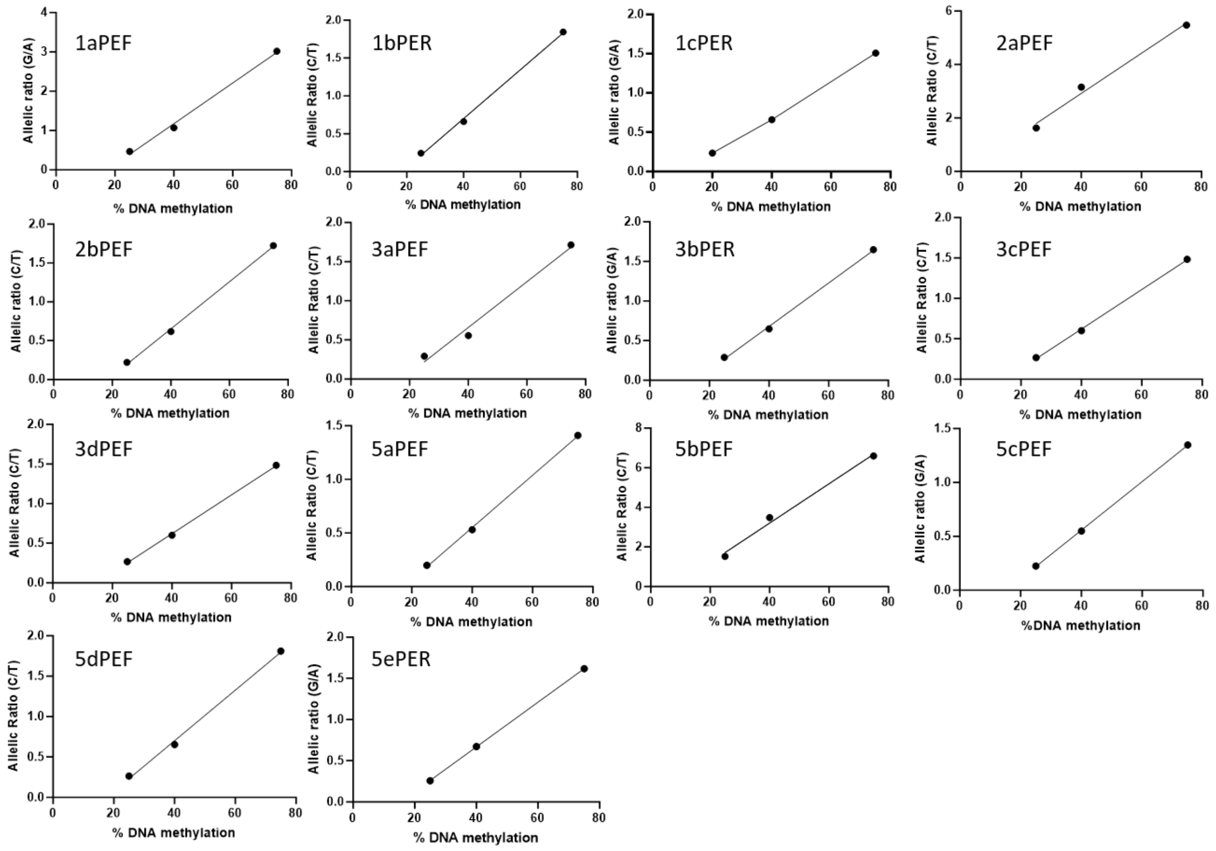

Figure S4. DNA methylation standard curves for the 14 CpG sites within the DRRα. Two CpG sites within amplicon 4 were excluded from further analysis because they were completely unmethylated in all samples. PEF and PER indicate the usage of forward or reverse SNaPshot primer, respectively.

Table S1. Sequence of primers used in this study

DNA methylation			
Amplicon	Genomic coordinate (hg38)	Sequence of PCR primer	Sequence of Snapshot Primer
Amp1	chr7:99692570-99692828	F: TATTTTTTGGAGGTTTTGTTTATTTTTT	1aPEF: TTTGATTATTGATATTTTTTTTTATAGTTGAT
		R: CACAAAATAACTAACAACCTTAAAAAA	1bPER: CGTAATACATACACAAACTTCAATAACC
			1cPER: AACTCAAAACCATAATACAAAAACTTC
Amp2	chr7:99692801-99693055	F: TTTTTTTAAGGTTGTTAGTTATTTTGTG	2aPEF: TTTTTTAGTTCGGATAAGTTTGTTAATAT
		R: ATCAAAAACCAAAAATAAATAAAACCAT	2bPEF: TTGGTGAGGAGTTGTGATTTTT
Amp3	chr7:99693142-99693435	F: AGTTAGATTTTTTAGTTGTAGGTTTTTT	3aPEF: TGGAAGTTTTATTTTAGAGGGGTATT
		R: CAACTCTAAAAAAAACAATAATTCTCTC	3bPER: AAAAACCACTTTCCAATAAAAACC
			3cPEF: TTAGGAATATATTTGAGGAGGTAGTTTATT
			3dPEF: TCTAAAAAAAACAATAATTCTCTCAACAC
Amp4	chr7:99693407-99693673	F: TGAGAGAATTATTGTTTTTTTTAGAGTT	4aPEF: TATAGAGGTAGTAGGTTTTGTTGAGTTG
		R: TACAACAATCTAAAATCCACCTAATATA	4bPER: ACTTACTATATAAACAAAAAAACCTAAAAACAC
Amp5	chr7:99693658-99693943	F: GATTTTAGATTGTTGTATTAGTAGTGAG	5aPEF: TTTAGATTGTTGTATTAGTAGTGAGTAAGTTTT
		R: AACTACCAACAAATCTAATTTCATTAAA	5bPEF: TTCGTGGGTGTGGTATTTGT
			5cPEF: CAAACCAAAAAATACCCTCCC
			5dPEF: GGAGTTGTAGATTAGAGTTGTTTTTATT
			5ePER: CAACTATTTTCAACCCCAATTAAAC
DNA methylation standard curve			
Primers used to amplify DRR region		DRRF: TGTTGGCCTGCTTTGCTAGTT	
		DRRR: ACAATACATGAAAAAGCAAAAAGGC	
Primers used for qPCR in iPSC and its derived cells and CRISPRi experiments			
gene-specific primers for cDNA synthesis			
		Sequence of primer	
	CYP3A4	GACAGAATAACATTCTTTCACTAGCACTG	
	CYP3A5	AAGACAGAATAACATTCTTTCACTAGCACT	
	CYP3A7	CTCCGGTTTGTGAAGACAGAATAAC	
SYBR Green-base qPCR			

		forward primer	reverse primer
	CYP3A4	CTCTCATCCCAGACTTGGCCA	ACAGGCTGTTGACCATCATAAAAG
	CYP3A5	GACCTCATCCCAAATTTGGCGG	CAGGGAGTTGACCTTCATACGTT
	CYP3A7	GATCTCATCCCAAACTTGGCCG	CATAGGCTGTTGACAGTCATAAATA
	b-actin	GAGAAGAGCTACGAGCTGCCT	GGTAGTTTCGTGGATGCCAC
CRISPRi gRNA sequences			
	NC	ACGGAGGCTAAGCGTCGCAA	
	DRRa	GCTGCAGCTTGATAGAGGAG	

Table S2. Basic statistics of the DNA methylation levels of the 16 CpG sites in 10 samples

Site	Mean	StDev	CoefVar	Minimum	Maximum	IQR
1a	33.09	10.98	33.18	25.59	61.32	5.88
1b	27.84	7.23	25.96	22.05	45.7	5.38
1c	28.78	8.13	28.26	21.16	48.73	5.53
2a	12.36	9.78	79.12	4.87	38.22	6.39
2b	56.28	10.38	18.43	43.26	77.6	13.64
3a	20.186	0.394	1.95	19.727	20.869	0.577
3b	33.32	3.43	10.28	26.5	37.62	6.11
3c	24.113	1.193	4.95	22.207	26.144	1.091
3d	26.652	1.001	3.75	25.038	27.876	1.692
4a	0	0	0	0	0	0
4b	0	0	0	0	0	0
5a	25.565	1.932	7.56	23.48	29.058	3.267
5b	10.38	0.601	5.79	9.44	11.162	1.238
5c	26.372	2.431	9.22	22.507	31.416	2.009
5d	27.04	5.95	22	13.22	36.67	4.41
5e	38.55	7.42	19.24	24.47	51.53	9.6

Table S3. Correlation between the DNA methylation levels of the 14 CpG sites in 10 samples. The correlation coefficient r of each pair is shown.

ID	1a	1b	1c	2a	2b	3a	3b	3c	3d	5a	5b	5c	5d
1b	0.993												
1c	0.985	0.994											
2a	0.882	0.888	0.89										
2b	-0.229	-0.2	-0.237	-0.346									
3a	0.112	0.184	0.167	-0.17	0.584								
3b	0.265	0.323	0.375	0.289	-0.151	0.207							
3c	0.748	0.727	0.758	0.71	-0.501	-0.558	0.193						
3d	0.61	0.631	0.66	0.761	-0.745	-0.358	0.418	0.774					
5a	0.727	0.792	0.769	0.66	-0.035	0.429	0.469	0.303	0.574				
5b	0.659	0.73	0.737	0.751	-0.166	0.234	0.694	0.371	0.675	0.889			
5c	0.703	0.766	0.805	0.604	-0.209	0.415	0.599	0.419	0.638	0.797	0.775		
5d	0.615	0.649	0.708	0.641	-0.155	0.035	0.771	0.637	0.608	0.537	0.701	0.769	
5e	0.628	0.632	0.681	0.54	-0.232	0.002	0.766	0.569	0.555	0.487	0.587	0.667	0.918

Table S4a. The composition of the CYP3A pools in the 246 liver samples.
The basic statistics for the percentage of each CYP3A in the total CYP3A pool is shown.

	Mean (%)	SD	Median (%)	Minimum (%)	Maximum (%)	Q1 (%)	Q3 (%)	IQR (%)
CYP3A4	81.96	18.33	87.46	7.03	99.27	76.06	95.07	19.02
CYP3A5	13.8	16.24	7.67	0.64	90.55	2.68	17.75	15.07
CYP3A7	4.24	7.91	0.99	0.01	55.65	0.28	4.01	3.72

Table S4b. The composition of the CYP3A pool in the 70 liver sample subset with DNA methylation data.
The basic statistics for the percentage of each CYP3A in the total CYP3A pool is shown.

	Mean	SD	Median	Minimum	Maximum	Q1	Q3	IQR
CYP3A4	77.83	20.75	83.34	8.37	99.27	69.31	93.69	24.38
CYP3A5	15.42	18.48	8.92	0.71	90.55	2.14	21.17	19.03
CYP3A7	6.76	11.31	1.52	0.02	55.66	0.56	7.86	7.29

Table S5. Changes in the expression levels of the CYP3As during iPSC to hepatocyte differentiation

	Expression level (log), Mean ± SD		
Time	CYP3A4	CYP3A5	CYP3A7
d0	0.63 ± 0.28	3.94 ± 0.07	0.93 ± 0.12
d5	1.02 ± 0.16	3.84 ± 0.13	1.63 ± 0.22
d10	1.59 ± 0.11	5.39 ± 0.16	2.23 ± 0.22
d21	2.43 ± 0.20	5.59 ± 0.05	3.22 ± 0.23
Liver	7.08 ± 0.72	6.08 ± 0.54	5.48 + 0.85

Table S6. Demographics of liver donors measured for DNA methylation at the DRR (n=70)

	All	AA	EA
	n=70	n=33	n=37
Age, years (mean ± SD)	57 ± 17	56 ± 19	58 ± 14
Female, n (%)	35 (50%)	16 (48%)	19 (51%)
